# Tuning the properties of magnetic thin films by interaction with periodic nanostructures

**DOI:** 10.3762/bjnano.3.93

**Published:** 2012-12-07

**Authors:** Ulf Wiedwald, Felix Haering, Stefan Nau, Carsten Schulze, Herbert Schletter, Denys Makarov, Alfred Plettl, Karsten Kuepper, Manfred Albrecht, Johannes Boneberg, Paul Ziemann

**Affiliations:** 1 Institut für Festkörperphysik, Universität Ulm, 89069 Ulm, Germany; 2 University of Basel, Department of Physics, 4056 Basel, Switzerland; 3 Institut für Physik, Technische Universität Chemnitz, 09107 Chemnitz, Germany; 4 IFW Dresden, Helmholtzstraße 20, 01069 Dresden, Germany; 5 Universität Osnabrück, Fachbereich Physik, 49069 Osnabrück, Germany; 6 Fachbereich Physik, Universität Konstanz, 78457 Konstanz, Germany

**Keywords:** colloidal lithography, magnetic data storage, magnetic nanostructures, percolated films

## Abstract

The most important limitation for a significant increase of the areal storage density in magnetic recording is the superparamagnetic effect. Below a critical grain size of the used CoCrPt exchange-decoupled granular films the information cannot be stored for a reasonable time (typically ten years) due to thermal fluctuations arbitrary flipping of the magnetization direction. An alternative approach that may provide higher storage densities is the use of so-called percolated media, in which defect structures are imprinted in an exchange-coupled magnetic film. Such percolated magnetic films are investigated in the present work. We employ preparation routes that are based on (i) self-assembly of Au nanoparticles and (ii) homogeneous size-reduction of self-assembled polystyrene particles. On such non-close-packed nanostructures thin Fe films or Co/Pt multilayers are grown with in-plane and out-of-plane easy axis of magnetization. The impact of the particles on the magnetic switching behavior is measured by both integral magnetometry and magnetic microscopy techniques. We observe enhanced coercive fields while the switching field distribution is broadened compared to thin-film reference samples. It appears possible to tailor the magnetic domain sizes down to the width of an unperturbed domain wall in a continuous film, and moreover, we observe pinning and nucleation at or close to the imprinted defect structures.

## Introduction

Perpendicular recording media currently in use consist of magnetic CoCrPt grains that are exchange-decoupled from each other by a thin layer of Si oxide or Ti oxide at the grain boundaries. A single magnetic bit is stored on the hard disk by magnetizing a small region consisting of several tens of magnetic grains with parallel magnetization either pointing up- or downwards. Increasing areal densities of recording bits, while maintaining the signal-to-noise ratio, demands a reduction of the grain size [1]. As a result of the miniaturization, however, the grain size approaches the superparamagnetic limit, below which stored information is lost due to thermal fluctuations of the magnetization. To some extent this problem can be circumvented by increasing magnetic anisotropy energy densities [2–8], but alternative approaches such as tilted magnetic recording [9], exchange-coupled composite media [10–11], exchange-spring media [12], or percolated perpendicular media [13–14] promise storage layers with significantly reduced transition noise at increased areal densities. In this contribution we focus on the latter approach: percolated magnetic films.

**Figure 1:**
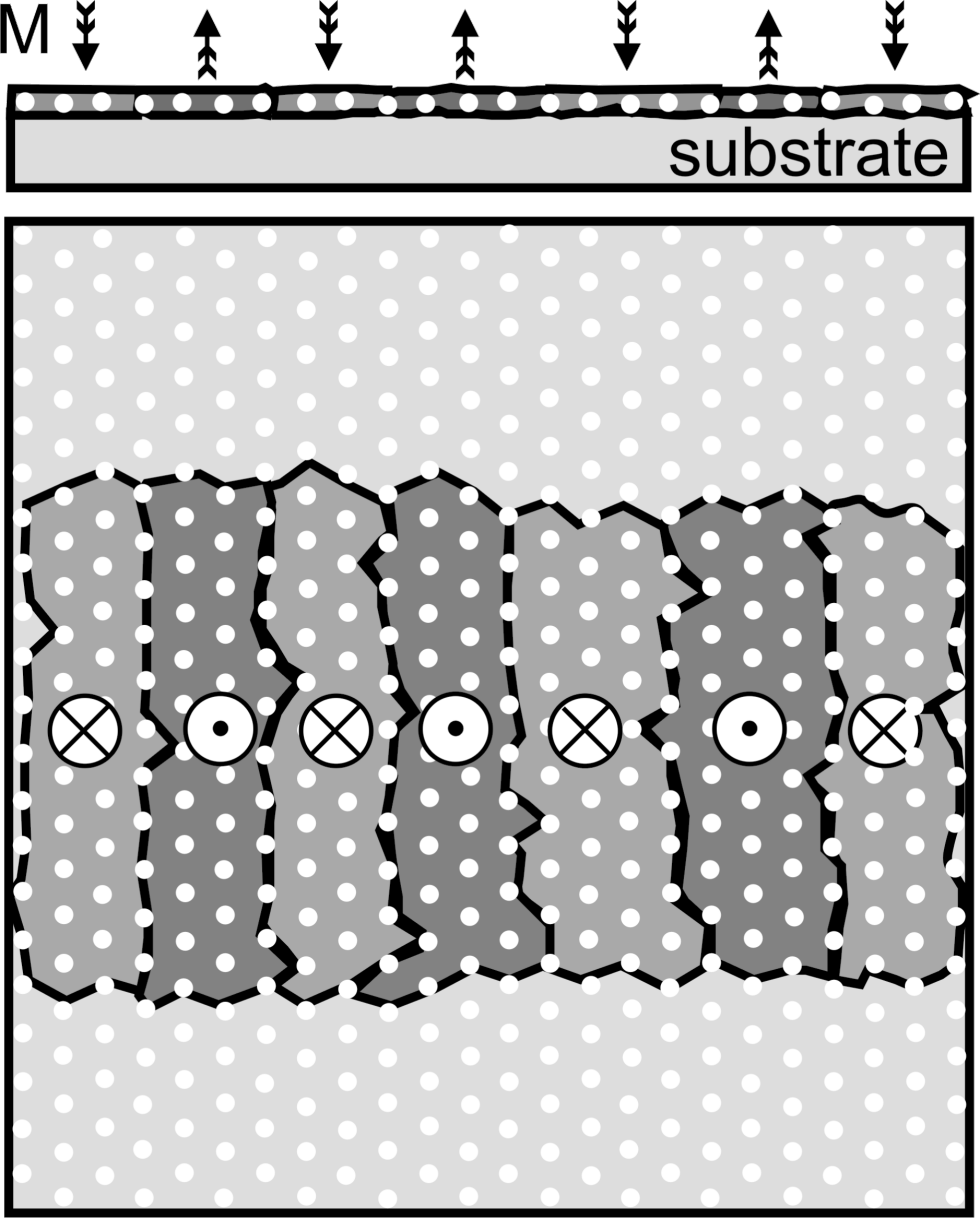
Principle of percolated perpendicular media. The exchange-coupled film is interspersed by nonmagnetic nanostructures (white dots) allowing domain-wall (black lines) pinning.

Such percolated media consist of exchange-coupled magnetic films with densely distributed pinning centers. In this case the magnetic stability is determined by the energy needed to dislodge a domain wall from its pinning site. The principle is illustrated in Figure 1. Domain walls are pinned at artificial defects imprinted by nanostructuring techniques. Defects may consist of an array of voids [15] or, even simpler, of nonmagnetic nanoobjects, such as particles, optimized with respect to both mutual distance and rather low filling factor. In that case, the information-storing layer is subsequently deposited onto the nanoobjects. In this way, in addition to the exchange-coupled film in between the objects, magnetic caps are formed on top of them. As a result, the achievable storage density is determined by the defect density.

Different preparation techniques have been tested for high defect densities, i.e., serial e-beam or ion-beam lithography, nanoperforated templates, polymer structures, or colloids, to name a few [15–18]. In the present contribution we have chosen a platform that allows the variation of the relevant parameters such as nanostructure size and their mutual distance, which are not easily accessible by the approaches mentioned above. The technique is based on self-assembly and homogeneous size reduction of polystyrene (PS) colloids [19] ending up with a non-close-packed monolayer of colloidal particles, which, in turn, serves as a template for the subsequent deposition of magnetic films.

In the following we first discuss the achievements and limitations of the technique. Then the tailoring of the magnetic switching process is presented for in-plane- and out-of-plane-magnetized films. Finally, an alternative approach towards smaller defect structures is introduced. We studied the magnetic reversal of Co/Pt multilayers with out-of-plane anisotropy deposited on a more densely packed array of Au nanoparticles.

## Results and Discussion

### Preparation of percolated magnetic thin films

**Figure 2:**
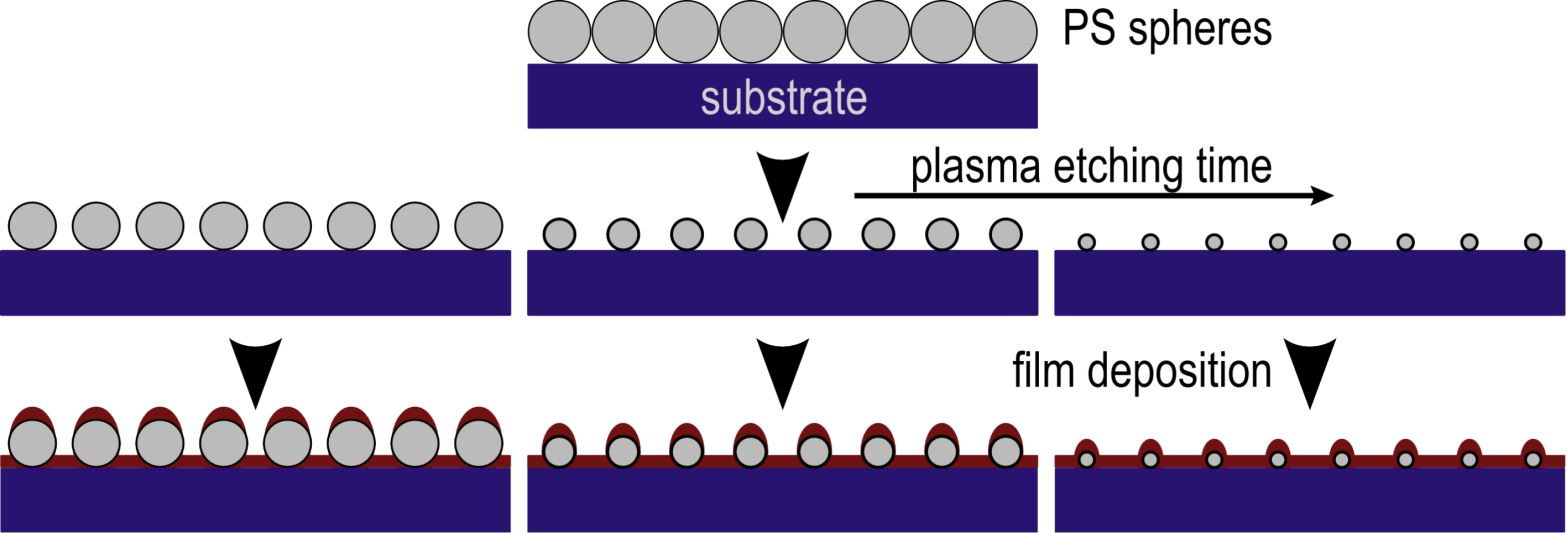
Schematics of the sample preparation. Self-assembled, close-packed monolayers of PS spheres are deposited on a substrate. Isotropic oxygen-plasma etching results in the homogeneous size reduction of PS particles. Finally, a magnetic film is deposited onto the substrates and capped by a thin Pt layer for oxidation protection.

Percolated magnetic thin films have been prepared by various techniques [15–16]. One necessary requirement is a homogeneous coverage of a given substrate on centimeter length scales. Such structures can be achieved by self-assembly of PS spheres [20] and subsequent plasma-assisted etching of these particles down to the targeted diameter [19]. This approach allows variations of the distance and the size of the nanostructure by choice of (commercially available) colloids and the adjustment of the remaining diameter by total etching time, respectively. Figure 2 presents a scheme illustrating the method for one initial diameter and three final sizes of PS spheres at constant distance achieved after different plasma etching times, as indicated by the arrow in Figure 2. Deposition parameters (thickness and material) of the magnetic film can then be chosen in such a way that intrinsic properties of the magnetic film result in a domain-wall width matching the distance of the nanostructure underneath. This interplay of nanostructuring and magnetism will be discussed in the following subsections. First, we present achievements and limitations of the approach.

Monolayers of PS spheres have been prepared by drop-drying [21], spin-coating [22], Langmuir–Blodgett deposition or dip-coating techniques [23]. For PS sizes of 200 nm and below it turned out experimentally that a homogeneous monolayer free of large spare, double or multilayer subregions (a prerequisite for integral magnetic characterization) can be achieved best by dip coating. For this purpose, Si substrates were used after hydrophilization in oxygen plasma, resulting in SiO_2_ layer thicknesses of about 5 nm. Commercial PS spheres [24] of average initial diameter of 190 or 95 nm were diluted to 1% w/v in purified water followed by dip-coating at a retraction velocity of typically 1 mm/min. Note that all parameters are slightly adjusted for each PS suspension. One general limitation is that the degree of hexagonal order of PS spheres becomes worse for smaller particle sizes due to the broadening of the relative size distributions of the colloids. This effect, however, plays a minor role in the context of the present work, since domain wall pinning, nucleation, and propagation are related to the nanostructure size and distance and in the simplest approximation not to the degree of hexagonal order.

**Figure 3:**
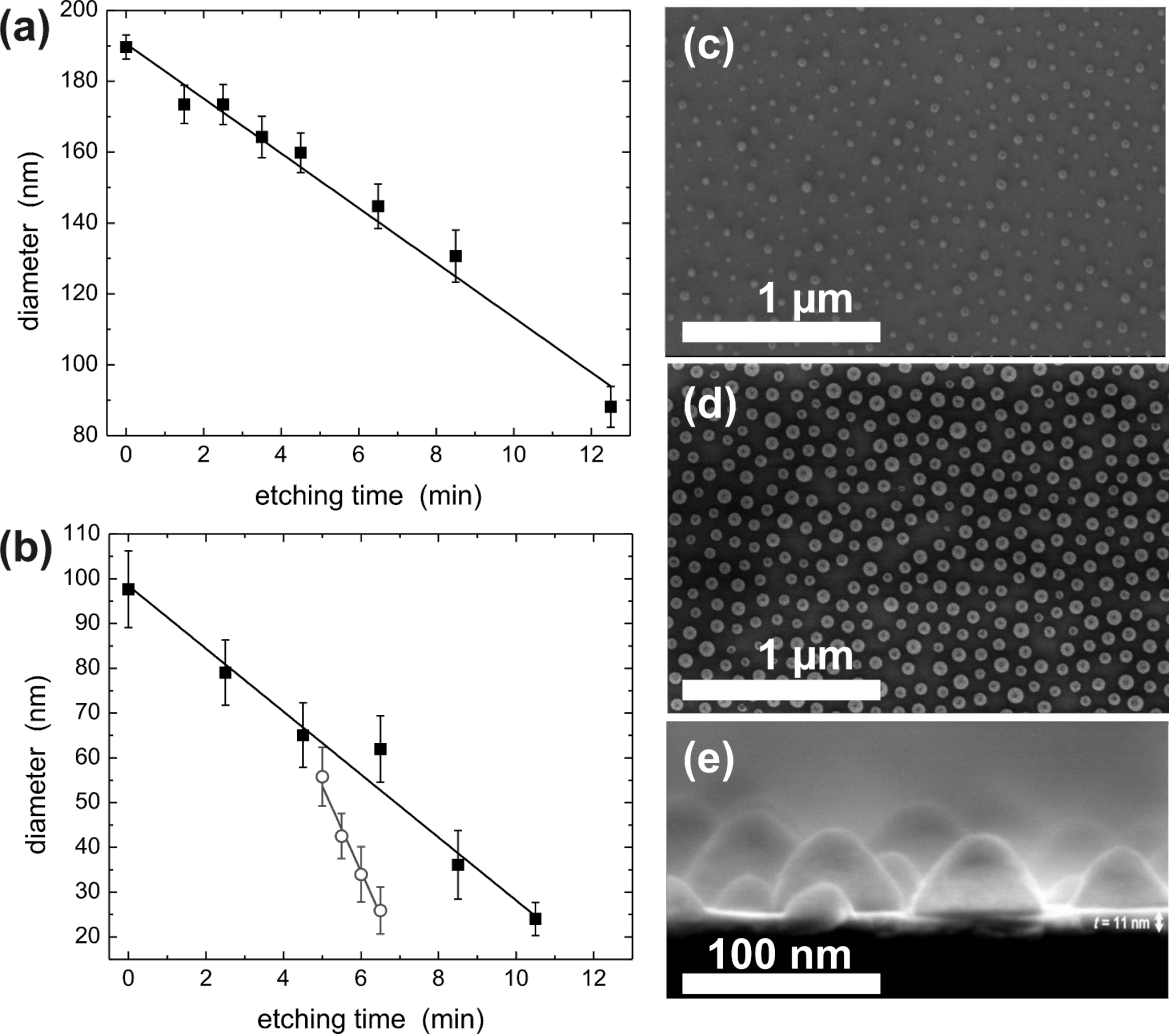
Size reduction of PS spheres as a function of etching time in isotropic oxygen plasma at ambient temperature for an initial particle diameter of (a) 190 ± 3 nm and (b) 95 ± 8 nm. Details are given in the text. Panels (c) and (d) show top-view SEM images of percolated Fe films deposited on size-reduced PS spheres of *d* = 35 ± 8 nm and *d* = 50 ± 8 nm at distance *a* = 95 ± 7 nm, respectively. The cross-section SEM image in (e) clearly reveals the magnetic-cap structure on PS spheres at film thickness *t* = 11 ± 1 nm on Si/SiO_2_ substrates. Panels (a) and (b) are reproduced with permission from reference [19] – Copyright 2009 Wiley.

The homogeneous size reduction of close-packed monolayers was performed by isotropic oxygen plasma etching at a constant pressure of about 7 Pa and ambient temperature. Details can be found elsewhere [19,25]. The applied conditions resulted in typical etching rates of 6–8 nm/min maintaining both the spherical shape and the initial position of the PS particles. Figure 3 presents results of the etching process for 190 and 95 nm particles as a function of etching time in panels (a) and (b), respectively. The diameter of particles constantly shrinks during the etching process as indicated by the linear fits. It is worth noting that the homogeneity of the etching process can be improved by an annealing step at 75 °C for 2 h at an intermediate diameter for surface smoothing, as shown by the circles in Figure 3b. This, however, enhances the etching rate by a factor of three. The etching technique is by no means restricted to 200 nm PS particles or below and can easily be adapted to larger initial sphere diameters, which are, however, not the focus of the present work.

After the preparation of the non-close-packed PS nanostructure, subsequent growth of magnetic films can be carried out in standard deposition chambers under ultrahigh-vacuum conditions. The percolated magnetic films discussed below were deposited either by pulsed laser ablation (Fe films) or e-beam evaporation (Co/Pt multilayers). For preparation details see, e.g., [25–26]. Panels (c) and (d) in Figure 3 show top-view scanning electron microscopy (SEM) images of Fe films (thickness *t* = 11 nm) deposited on top of *d* = 35 ± 8 nm and *d* = 50 ± 8 nm particles at a distance of *a* = 95 ± 7 nm, respectively. Note that 1–3 nm Pt capping layers were used for oxidation protection. PS particles can be clearly seen while the Fe film exhibits contrast preferentially in cross-section SEM images. The cross-section SEM image Figure 3e, however, unambiguously reveals the morphology of the percolated Fe film. While between the 35 nm particles a smooth film grows on Si/SiO_2_ substrates, Fe caps can be easily identified. Remarkably, Fe caps are in contact with the film between the colloidal spheres, although the deposited film thickness (11 nm) is small compared to the average particle diameter of *d* = 35 nm. Note that PS spheres including the magnetic caps on top may also be removed by chemo-mechanical polishing leading to a void structure, which may potentially be used as 2-D artificial spin-ice systems [27]. In the following, however, we focus on percolated films with magnetic caps present. In summary, the described fabrication delivers a percolated magnetic film, from which strongly modified magnetic properties can be expected as compared to an unstructured reference film. Magnetic studies following the above approach are presented for in-plane- and out-of-plane-magnetized films in the next two subsections, respectively.

**Figure 4:**
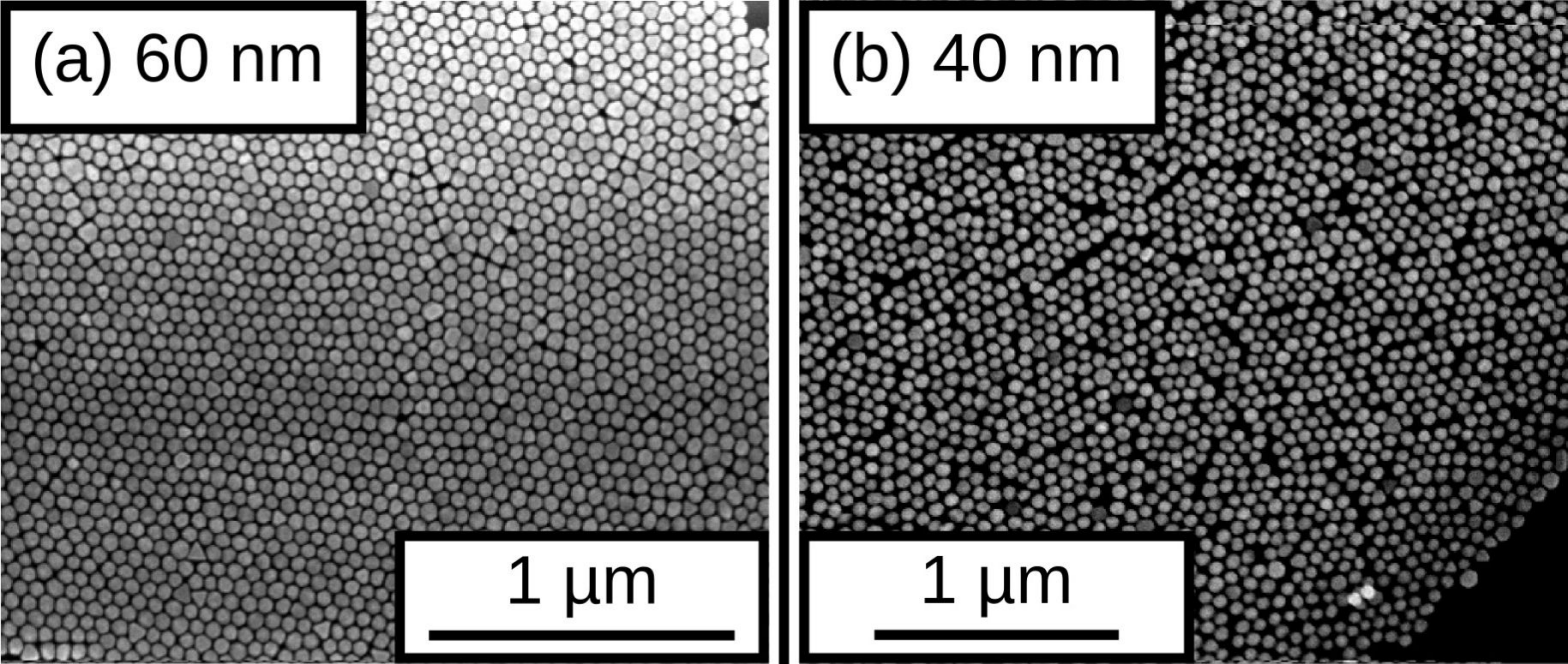
SEM images of monolayer assemblies of commercial Au nanoparticles with (a) 60 nm diameter and (b) 40 nm diameter on Si/SiO_2_ substrates.

Alternatively to film deposition on size-reduced PS particles for percolated magnetic films, one may use self-assembly of inorganic spheres, such as Au nanoparticles, for nanostructuring. Commercially available Au colloid solutions with particle sizes of 60 and 40 nm have been used to prepare monolayers of self-assembled Au particles on thermally oxidized Si(100) substrates [28]. The Au colloid solution was diluted with pure ethanol in the volume ratio of 2:1. A volume of 60 µL of such solution was dispersed onto the substrates and dried under ambient conditions in a covered box to prevent air flow (see [29] for details). The arrangement of the nanoparticles in the islands was investigated by SEM. Due to the low particle concentration in the colloid solution they form a number of irregularly shaped islands, which extend over several tens of microns. Figure 4 presents two SEM micrographs of such samples with Au nanoparticle arrangements with particle diameters of 60 and 40 nm, respectively. The 60 nm sized particles arrange in a hexagonal close-packed order, only disturbed by particles of slightly different size. The 40 nm particles do not order as well as those with 60 nm diameter. Voids with sizes of about 10 to 20 nm are formed in between Au particles. On the one hand, this approach allows significant reduction of the average distance of nanostructures as compared to the PS colloids; on the other hand the homogeneity of coverage is strongly reduced. The impact of these differences on the magnetic behavior of the percolated film is discussed in the following sections.

After preparation, the Au particle assemblies were introduced in a molecular beam epitaxy (MBE) chamber equipped with an e-beam evaporator loaded with Co and Pt (purity greater than 99.99%). The multilayer stack [Co(0.3 nm)/Pt(0.8 nm)]_8_ was deposited on a 5 nm thick Pt buffer layer. An additional 3 nm thick Pt cover layer was deposited to protect the samples from oxidation. The deposition of the metal films was performed simultaneously on particle assemblies as well as on planar substrates for reference.

### Magnetic switching in percolated Fe films

**Figure 5:**
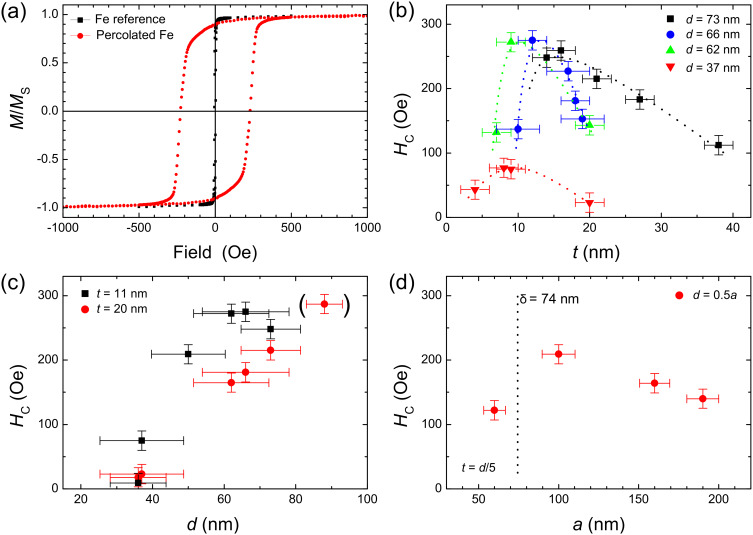
(a) Magnetic hysteresis loops of a percolated Fe film (*a* = 95 nm, *d* = 66 nm, *t* = 17 nm) and a thin-film reference sample taken in the in-plane geometry at ambient temperature. Panel (b) shows the experimental coercive field as a function of Fe layer thickness *t* for different diameters *d* of PS spheres at a distance *a* = 95 nm. Dotted lines are guides to the eye. In (c) *H*_C_ is presented as a function of *d* at *a* = 95 nm for two layer thicknesses *t*. In panel (d) the coercive field is displayed as a function of the sample periodicity *a* for PS sphere diameter *d* = 0.5*a* and optimized Fe film thickness *t* = *d*/5. Additionally, the experimentally determined domain wall width δ = 74 nm of continuous Fe films is marked.

The impact of such percolated structures as a function of thickness *t*, the remaining diameter of particles *d* and the average center-to-center distance *a* on the magnetic reversal was investigated by integral superconducting quantum interference device (SQUID) magnetometry. Figure 5a compares in-plane hysteresis loops of a percolated film (*a* = 95 nm, *d* = 66 nm, *t* = 17 nm) and a simultaneously prepared thin-film (*t* = 17 nm) reference sample on Si/SiO_2_ substrate at ambient temperature. While the thin Fe film has a narrow and steep hysteresis with high remanent magnetization with respect to the saturation magnetization, and small coercive field, the percolated Fe film of identical thickness exhibits a strongly enhanced coercive field of *H*_C_ = 250 Oe. Moreover, the hysteresis is more S-shaped with slightly reduced remanent magnetization and large saturation field of about 3000 Oe as compared to the reference. Such behavior can be attributed to domain wall pinning, presumably at the locations of the particles. For deeper insights into the switching process, however, it is necessary to apply magnetic microscopy techniques, as discussed below.

Using integral characterization techniques, such as SQUID magnetometry, it is convenient to determine the coercive field *H*_C_ as a function of the geometric parameters *t*, *d*, and *a* of the samples, as shown in Figure 5b–d, respectively. In panel (b) *H*_C_ is displayed as a function of the film thickness *t* for different remaining particle diameters. All samples were prepared from PS spheres with an initial diameter of *a* = 95 nm. The coercive field is found to be larger in all samples compared to the reference films having *H*_C_ < 10 Oe. It is apparent that *H*_C_ has a gradually shifting maximum moving from about *t* = 10 nm for *d* = 37 nm towards *t* = 16 nm for *d* = 73 nm. This indicates that the optimum pinning effect has to be adjusted for each individual set of parameters *t*, *d*, and *a*. The appearance of a maximum *H*_C_ can be explained as follows: For small thicknesses *t* the samples consist of films with non-magnetic holes inside and exchange-decoupled Fe caps on top of the PS particles. In this limit the cap structures have a minor influence on the integral magnetic reversal behavior of the film. Consequently, the switching is dominated by the film reversal, where the small holes, which eventually pin domain walls, will lead to an enhancement of the coercive field to *H*_C_ = 45 Oe as compared to the reference. Above a certain thickness, film and caps form an exchanged-coupled magnetic structure as shown in the cross-section SEM image in Figure 3e. In this case, domain-wall pinning is expected to be reduced. For larger particle diameters a larger film thickness *t* is necessary to grow an exchanged-coupled percolated sample. In this way the rise and the shift of *H*_C_ can be explained qualitatively. For thicker films, however, the sample morphology appreciably changes towards a rippled, but rather continuous film leading to decreased coercive fields. The pinning effect is further investigated as a function of particle diameter *d* for two thicknesses *t* = 11 nm and *t* = 20 nm as shown in Figure 5c. We observe a gradual increase of *H*_C_ up to 280 Oe for *t* = 11 nm, while *H*_C_ of the thicker film lags behind probably due to the minor impact of thicker films on the magnetic switching, as discussed above.

The strongest influence of the imprinted nanostructures on the magnetic switching behavior may be expected when the diameter and/or distance matches a magnetic length scale such as the domain wall width of unperturbed films. In such a case, growing domains would experience the largest resistance while for even smaller periods a strongly distorted domain pattern can be expected. To elucidate such correlations we vary the interparticle distance *a* while etching their diameter to *d* = *a*/2 and adjust the film thickness to *t* = *d*/5. Parameters were chosen in such a way as to guarantee a pinning effect close to its maximum as expected from Figure 5b. The results are shown in Figure 5d for periods between 60 and 180 nm. Although only a limited number of experimental points are available due to the complicated sample processing in this case, we expect the highest coercive field for this set of parameters in the interval between 60 and 100 nm. This finding can be compared to the experimentally determined, unperturbed Fe domain wall width as measured by scanning transmission X-ray microscopy (STXM).

For a deeper understanding of the micromagnetism, selected samples were investigated by STXM at the PolLux beamline at the Swiss Light Source, Paul-Scherrer-Institute in Villigen, Switzerland. The setup has been presented in detail elsewhere [30]. Percolated Fe films were prepared on commercial Si_3_N_4_ membranes (thickness 100 nm) in-line with the description for Si/SiO_2_ substrates above. Two Fe-L_3_ images were measured by scanning the sample with left and right circularly polarized light. The ratio of the two images reveals the magnetic domain structures at a resolution of better than 25 nm at the PolLux beamline. Sensitivity to in-plane magnetization of Fe films in the remanent state was achieved by tilting the sample normal by 30° with respect to the propagation direction of X-rays. Before imaging at ambient temperature, the samples were demagnetized by decreasing alternating fields in the horizontal in-plane direction.

A small fraction (a few percent) of the surface that is free of particles is typically found on samples prepared by dip-coating. In these areas the domain pattern of unperturbed films can be directly measured on one and the same sample. While typical domain sizes are in the range of 5–10 µm^2^, we determined the domain wall width δ of a continuous Fe film as δ = 74 ± 21 nm by a number of line scans across a domain wall of maximum contrast (not shown). This value is in good agreement with the thickness of the Bloch domain wall in Fe, which is about 80 nm. Furthermore, the thickness of the domain wall corresponds to the estimated period *a* = 60–100 nm in percolated Fe films (with 5*t* = *d* and 2*d* = *a*) giving a strong hint to the correlation of the distance between the nanostructures with the intrinsic domain wall width δ for the largest coercive fields due to magnetic pinning.

**Figure 6:**
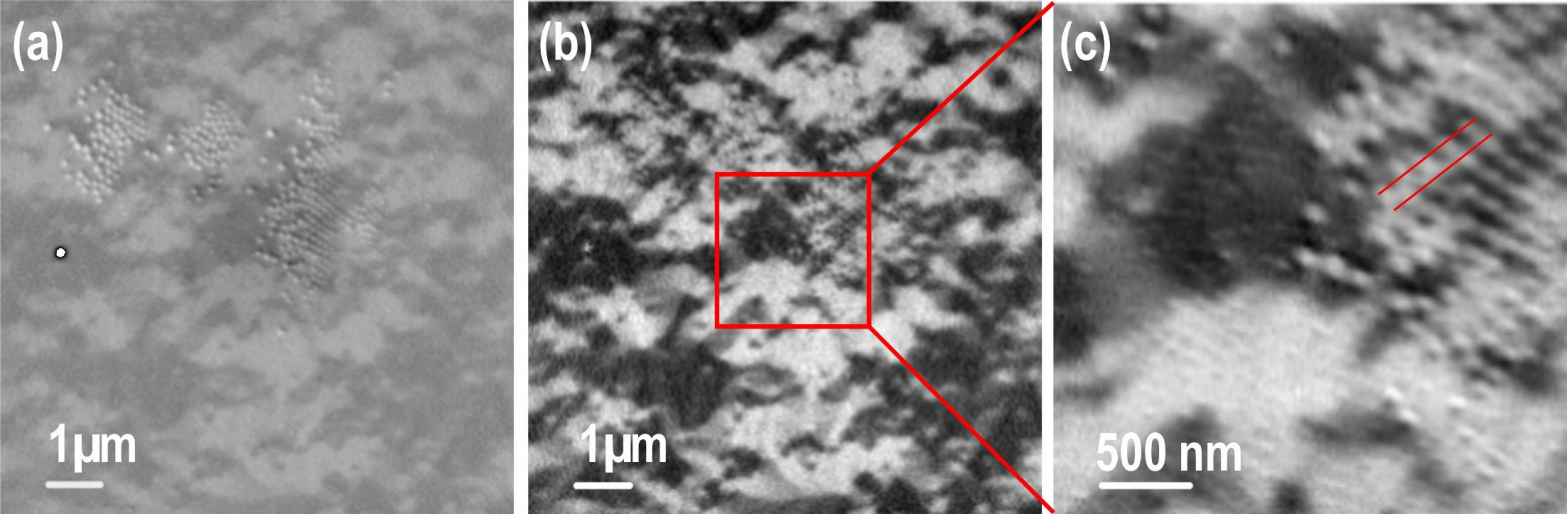
(a) Scanning transmission X-ray microscopy images of Fe films taken with right circularly polarized light at the Fe-L_3_ edge show PS particles as well as magnetic contrast in the film after sample demagnetization. Panel (b) presents the XMCD image of the identical sample spot. In (c) an XMCD image at higher resolution is shown revealing two regions (lower left and top right) of different PS particle diameters. The underlying domain sizes appear strongly varied in these regions.

The magnetic domain pattern was measured in more detail for a percolated Fe film with nominally *a* = 95 nm, *d* = 35 nm, *t* = 18 nm. The parameters were chosen in such a way that the maximum field available in the setup (300 Oe) was sufficient to switch the magnetization of the sample. Figure 6a presents a single STXM image taken with right circularly polarized light. Some particles are clearly visible in the upper part of the sample while magnetic contrast of domains is directly observable. Enhancement of the magnetic signal can be achieved by division of STXM images taken with opposite helicities of X-rays at the price of losing the particle contrast almost completely. This is demonstrated in Figure 6b. The magnification in Figure 6c reveals the actual sample morphology. Starting on the lower left of the image the faint contrast visible in single domains is produced by 35 nm particles at *a* = 95 nm while on the upper right some hundred larger particles have self-assembled with a period of 150–170 nm. In our experiments, such demixing effects of a small contamination of larger particles are often observed during self-assembly. The fraction of larger particles was estimated by large-scale SEM imaging and was found to be well below 0.1%. Taking equal etching rates for the 95 nm and approximately 160 nm particles, as suggested by the data presented in Figure 3, the upper right area reveals the ratio *d*/*a* = 100 nm/160 nm = 0.63 while in the lower left area we set *d*/*a* = 0.37. Based on the drastic difference in the diameter-to-period ratio and the results shown in Figure 5 we expect a much stronger pinning effect of the percolated film at larger *d*/*a* ratio. In the extreme, this may result in domain patterns with a minimum size as small as the distance between two particles. This feature is observed in Figure 6 indicated by the parallel red lines.

### Magnetic switching in percolated perpendicular media

The concept of percolated perpendicular media is tested by film deposition onto size-reduced PS particles with *a* = 180 nm and *d* = 40 nm and a planar reference sample. Note that for such small nanostructures prepared from particles initially spaced at 180 nm the spherical shape is not maintained and rather semispheres are formed on the Si/SiO_2_ substrate [25]. A [Co(0.3 nm)/Pt(0.8 nm)]_12_ multilayer stack with an effective perpendicular magnetic anisotropy of 0.3 MJ/m^3^ [31] has been deposited by alternating evaporation from pure Co and Pt sources by e-beam evaporation under ultrahigh vacuum conditions at ambient temperature. For integral characterization polar magneto-optical Kerr effect (MOKE) magnetometry with a spot size of about 100 µm was used to investigate magnetic switching (details are given in reference [25]). For this sample, with structure sizes of 40 nm at a distance of 180 nm, the contribution of magnetic caps to the MOKE signal is small due to the reduced filling factor and the stronger scattering of the reflected laser beam off the particle caps. The planar reference film shows a sharp switching at a coercive field of µ_0_·*H*_C_ = 290 mT and full remanent magnetization, indicating a reversal process dominated by the nucleation and subsequent propagation of domain walls [32].

**Figure 7:**
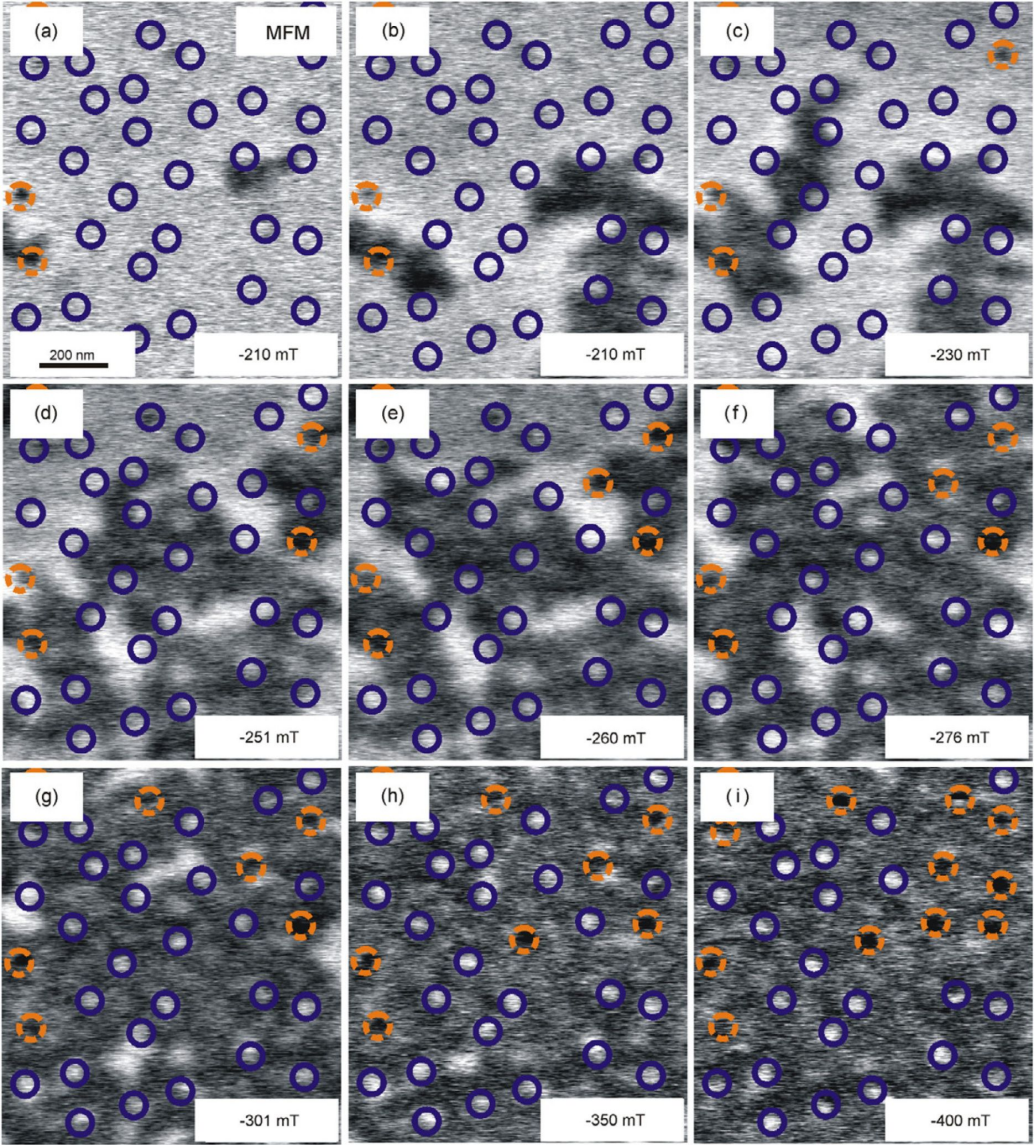
(a–i) MFM images of the same spot taken in different perpendicular fields after driving the sample to the positive saturated state at +1.6 T. Switched magnetic caps are indicated by dashed orange circles, while purple circles mark the unaffected caps. Panels (a) and (b) are two scans at identical field showing the growth of nucleated domains in the direction of the external field. This process is either thermally activated or induced by the tip. The figure has been reproduced with permission from [25] – Copyright 2009 Institute of Physics.

The domain pattern of the sample has been recorded by atomic and magnetic force microscopy (AFM and MFM) after demagnetization in a perpendicular field. While in AFM mode the 40 nm dots were clearly resolved, the magnetic structure of the Co/Pt multilayer showed domain sizes of about 200 nm. Moreover, every single cap structure is in a single-domain state and decoupled from the film in between the particles. It turned out that magnetic domain walls correspond to the positions of the particles, this being the energetically favored configuration. For deeper understanding of the magnetic reversal process, in-field MFM in a custom-built setup using the constant-height mode was employed [33]. Figure 7 presents the switching process after saturating the sample in positive field perpendicular to the sample plane. The particle positions are displayed by circles as provided by an AFM prescan. The color code shows whether the particle cap has switched (orange) or not (purple). It is obvious that the nucleation of domains starts at about −210 mT close to the particle positions as displayed in Figure 7a and Figure 7b. Interestingly, the first magnetic caps already switch at this low field. The reversal mode of the film, however, is still dominated by domain-wall propagation. This can be easily identified when tracking the reversed areas in Figure 7b–g, and the domain walls preferentially stop at or close to the particle positions, i.e., domain walls are pinned at the defect sites. We do not observe strong exchange coupling between film and caps, since the majority of caps still remains in the initial state while the film is completely reversed (cf. Figure 7i). Thus, it is likely that magnetostatic interactions are important here leading to a strongly broadened switching field distribution (SFD) and a significantly enhanced coercive field. The most important contribution to the SFD is the nucleation field of individual magnetic caps, which is defined by its local magnetic anisotropy, size and shape. Thus, any size distribution of particles directly leads to the broadening of the SFD. From additional MFM investigations as a function of the external field, the hysteresis loops of the magnetic caps and the film can be separated by counting the number of switched caps and calculating the ratio of bright and dark contrast in the film region (see reference [25] for details). For the magnetic caps, this approach yields µ_0_·*H*_C_ = 450 mT and a broad SFD around 500 mT for the sample shown in Figure 7. Moreover, due to the low filling factor of defects, one expects that the MFM-extracted film hysteresis loop is similar to the hysteresis measured by integral techniques such as MOKE. Indeed, this has been demonstrated in reference [25].

### Magnetization reversal in arrays of spherical gold particles capped with Co/Pt multilayers

An alternative approach towards realizing percolated perpendicular media is the deposition of magnetic films with out-of-plane anisotropy on a densely packed array of hard spheres. This method was tested by growing a [Co(0.3 nm)/Pt(0.8 nm)]_8_ multilayer stack (sandwiched between 5 nm Pt buffer layer and a 3 nm Pt capping layer) on commercially available Au nanoparticles as described above.

**Figure 8:**
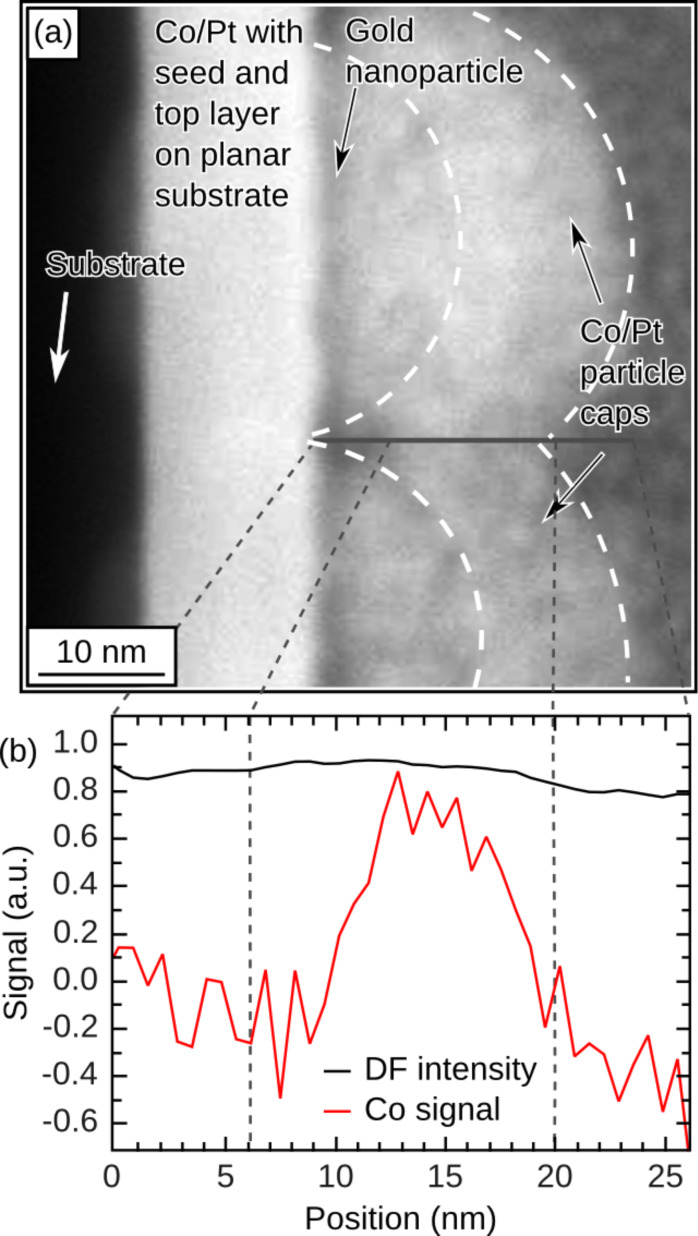
(a) DF-STEM image of 40 nm Au nanoparticles capped with a magnetic Co/Pt multilayer. Panel (b) shows an EELS line scan taken at the contact region between the two particles along the solid line shown in (a). The figure has been reproduced with permission from reference [29] – Copyright 2009 EUCEMAN.

The structure of the magnetic multilayer on the nanoparticles strongly influences its magnetic properties. By means of SEM and cross-sectional scanning transmission electron microscopy (STEM) investigations, it was found that, independent of the particle size, the multilayer (with a nominal thickness of 16.8 nm) follows the curvature of the particle array (Figure 8a). By electron energy loss spectroscopy (EELS) along the line displayed in Figure 8a, the Co content was probed by using intensity profiling of the Co-L_3,2_ edges. Figure 8b shows the Co signal, as well as the dark field (DF) intensity along the line scan. From the EELS line scan, Co is found to be present in the film material filling the contact region between the neighboring particle caps, suggesting that those caps are magnetically connected across the contact region, and form a continuous magnetic layer on top of the particle array [29]. Although no quantitative analysis of the amount of Co at the interconnection region was possible, exchange coupling between the particle caps is expected even if the multilayer structure of the Co/Pt film were disturbed, since Co–Pt alloys are ferromagnetic at room temperature down to a Co concentration of about 15 atom % [34].

**Figure 9:**
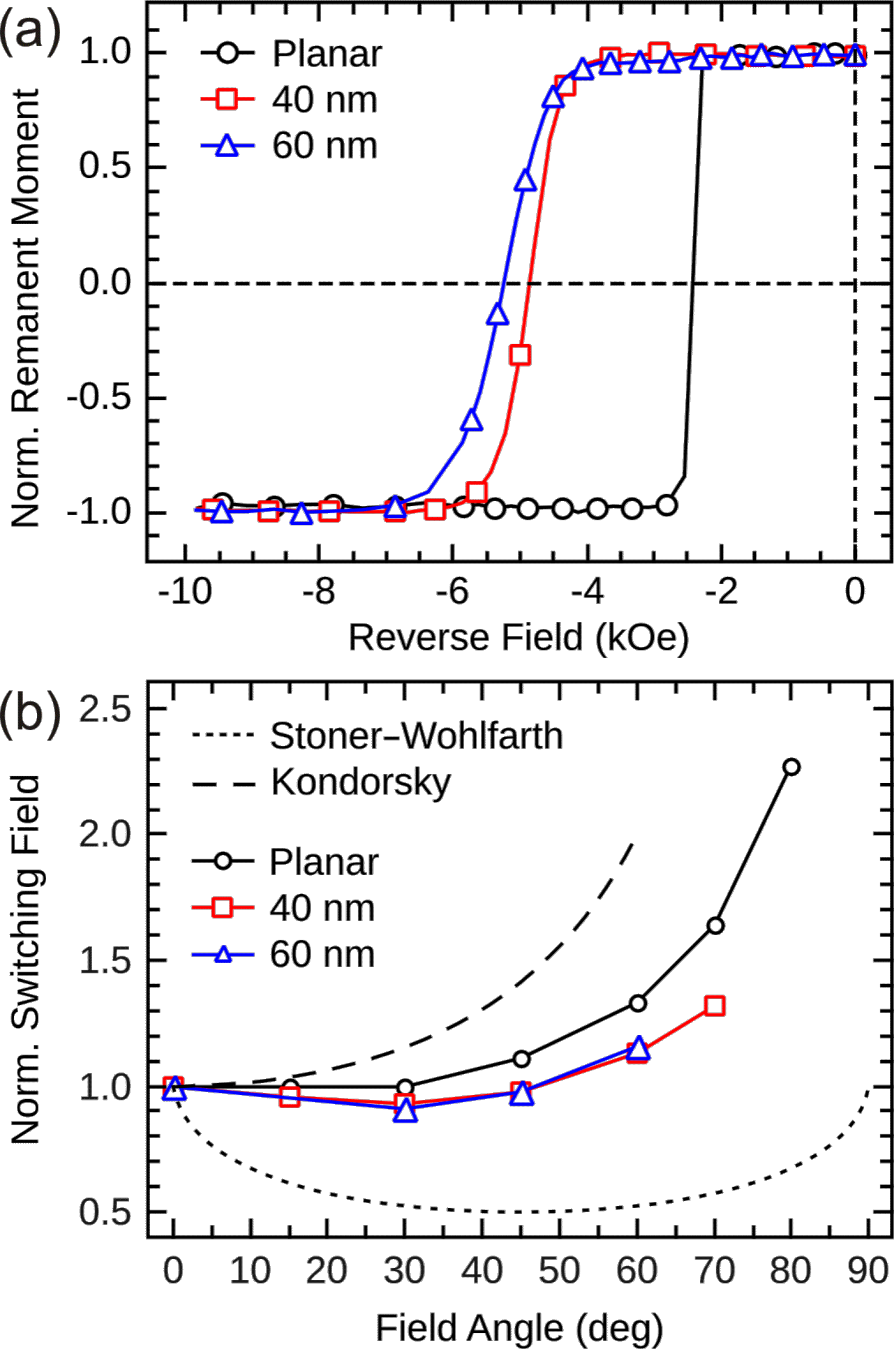
(a) MOKE remanence curves for Co/Pt multilayers grown on a planar substrate as well as on arrays of Au nanoparticles of 40 and 60 nm diameter. (b) Angular dependence of the switching field for the samples. The values beyond 60° for 60 nm particles and beyond 70° for 40 nm particles are not plotted, since the samples were not saturated in the available field. The figure has been reproduced with permission from reference [29] – Copyright 2009 EUCEMAN.

MOKE remanence curves were measured by saturating the sample, subsequently applying a reverse field and recording the remanent magnetization (Figure 9). The angle θ of the external magnetic field with respect to the sample normal was varied between 0 (perpendicular to the substrate plane) and 90° (in the substrate plane) without changing the polar detection geometry, thus allowing an angular-dependent study of the integral magnetic signal. The remanence curves taken on the samples at a field angle θ = 0° show that the switching field of the reference sample is as large as 2.4 kOe with rather narrow SFD. The switching field is substantially increased for the multilayers grown on nanoparticle arrays, and the SFD is significantly broadened compared to the reference sample. Considering the morphology of the magnetic film on the nearly densely-packed Au particles, one can understand this finding qualitatively: the rims around the particles act as sites for preferential domain-wall nucleation, while the pinning effect rather occurs in between particles, acting as defects here. The interplay of both effects may lead to the local stabilization of a magnetic domain in an individual particle cap. The broadening of the SFD is mainly attributed to the particle size distribution as well as to the magneto-static coupling between the neighboring caps in the array [35–36].

Increasing the angle θ results in a gradual increase of the switching field of the reference sample (Figure 9b) as expected for a strongly exchange-coupled magnetic film according to Kondorsky's model of depinning 180° domain walls (dashed line) [37]. The magnetic caps on arrays of 40 and 60 nm particles are found to be single-domain [29]. For a small, homogeneously magnetized nanostructure in a single-domain state, a coherent rotation process with a Stoner–Wohlfarth angular dependence [38] is expected (dotted line in Figure 9b). The magnetic caps, however, show only a weak angular dependence of the switching field with a small increase at high angles. This deviation can be understood, in part, by taking into account the distribution of easy axes within the nanocaps following the surface curvature. This necessarily leads to a rather incoherent magnetization reversal process in an individual cap [39], which has also been demonstrated by micromagnetic simulations [32].

## Conclusion

On the way towards alternative magnetic storage media, we have tested possible realizations of percolated media, consisting of magnetic films on top of periodic nanostructures. Such structures, realized by particle self-assembly techniques are able to effectively pin magnetic domains at the imprinted particle-induced defect structures. We generally observe enhanced coercive fields while the switching-field distribution is broadened compared to their continuous film counterparts.

In the first part of this contribution we presented results on nanostructuring techniques such as the self-assembly of PS particles and subsequent plasma etching ending up with well-separated nanostructures with adjustable sizes and distances. A linear dependence of the particle size with etching time has been observed down to particle sizes of 25 nm starting from initially 95 and 190 nm PS particles, respectively. Alternatively, the Au nanoparticles deposited on planar substrates may act as a nanostructuring material, which generally allows smaller distances of nanostructures, albeit with strongly reduced variability of diameter and interparticle distance.

On these nanostructures we deposited magnetic thin films with in-plane (Fe) and out-of-plane (Co/Pt multilayers) orientation of the magnetization under UHV conditions, respectively. Thereby, the film thicknesses have been adjusted to the height of the nanoparticles or below leading to an exchange-coupled film with magnetic cap structures grown on the particles in contact with the film. For thin Fe films, studies of the integral magnetic hysteresis as a function of film thickness, distance and size of the particles have demonstrated that strong magnetic pinning can be established at ambient temperature. In the extreme, we find a strongly enhanced coercive field *H*_C_ up to 280 Oe compared to Fe thin-film reference samples of identical thickness (*H*_C_ < 10 Oe). Further studies of the domain configuration by scanning transmission X-ray microscopy have provided first evidence that the particles (with magnetic caps) effectively decrease the domain size, if the distance, diameter, film thickness and magnetization are properly chosen. In this way it is possible to corral an individual magnetic domain in between a number of neighboring dots. Note that the optimum parameters are not completely independent and the strongest magnetic pinning cannot be unequivocally predicted on the basis of the presented experiments, since exchange-coupling to the magnetic caps is critical and difficult to control.

In a second study, we investigated the impact of such size-reduced colloidal nanostructures on film/particle systems with out-of-plane anisotropy. The magnetic reversal mode was strongly altered compared to the thin film reference. By detailed MFM investigations we have shown that magnetic domains first nucleate at particle positions and propagate further until pinning eventually occurs at the next particle position, i.e., a defect in the film morphology. Thus, this feature can be exploited to limit the domain size in between a lateral arrangement of defects on the nanoscale, which is the essential requirement for realizing percolated perpendicular media. However, the density of particles still has to increase towards periodic structures with interparticle distances below 20 nm. In turn, hard magnetic alloys such as FePt or CoPt have to be used for smaller defect periods.

Such a further reduction of size has been tested by self-assembly of 40 nm Au nanoparticles and subsequent deposition of Co/Pt multilayer films on top. Although magnetic exchange coupling between the caps in the array is expected, the magnetic caps are found to be in a single-domain state. In this respect, it is very likely that the differences in activation volume for nucleation and depinning of domain walls at defect sites induced by the underlying particle template alter the magnetic reversal mechanism.

In summary, the applied nanostructuring methods have proven very effective in limiting domain sizes by pinning domain walls at defect structures for both in-plane- and out-of-plane-magnetized thin films, as demonstrated by integral magnetometry and magnetic microscopies. The individual, local switching field can be tuned by adjusting the dimensions of the nanostructures and the film parameters. However, it turned out that nearly perfect homogeneity is necessary to achieve a narrow and predictable switching-field distribution. Further reduction of the nanostructure dimensions and the use of highly anisotropic materials are necessary to be competitive with storage media employing percolated perpendicular media.
